# Self-Isolation Due to COVID-19 Is Linked to Small One-Year Changes in Depression, Sleepiness, and Insomnia: Results from a Clinic for Sleep Disorders in Shiga Prefecture, Japan

**DOI:** 10.3390/ijerph17238971

**Published:** 2020-12-02

**Authors:** Ayaka Ubara, Yukiyoshi Sumi, Kazuki Ito, Arichika Matsuda, Masahiro Matsuo, Towa Miyamoto, Hiroshi Kadotani

**Affiliations:** 1 Graduate School of Psychology, Doshisha University, 1-3 Tatara Miyakodani, Kyotanabei, Kyoto 610-0394, Japan; cykc1005@mail2.doshisha.ac.jp; 2 JSPS Research Fellowships, 5-3-1 Kojimachi, Chiyoda-ku, Tokyo 102-0083, Japan; 3 Department of Sleep and Behavioral Sciences, Shiga University of Medical Science, Seta Tsukinowa-cho, Otsu, Shiga 520-2192, Japan; momo3@belle.shiga-med.ac.jp (K.I.); arichika@belle.shiga-med.ac.jp (A.M.); 4 Department of Psychiatry, Shiga University of Medical Science, Seta Tsukinowa-cho, Otsu, Shiga 520-2192, Japan; elasticvisco@gmail.com (Y.S.); mazzuo@belle.shiga-med.ac.jp (M.M.); twmymt@belle.shiga-med.ac.jp (T.M.); 5 Department of Anesthesiology, Shiga University of Medical Science, Seta Tsukinowa-cho, Otsu, Shiga 520-2192, Japan

**Keywords:** COVID-19, depression, insomnia, Japan, lockdown, self-isolation, sleep disorders, self-report, sleep duration, sleepiness

## Abstract

We aimed to analyze (a) the changes in depression, sleepiness, insomnia, and sleep habits in relation to the degree of self-isolation and (b) the effects of changes in sleep habits and social interactions on depression, insomnia, and sleepiness during the coronavirus disease 2019 (COVID-19) pandemic. We enrolled 164 patients who visited the sleep outpatient clinic in Shiga University of Medical Science Hospital. We compared the sleep habits, depression (Patient Health Questionnaire-9: PHQ-9), insomnia (Athens Insomnia Scale: AIS), and sleepiness (Epworth Sleepiness Scale: ESS) of patients during the period from April to July 2019 vs. May 2020 (a period of self-isolation due to COVID-19). A Wilcoxon signed-rank test indicated no significant differences in PHQ-9, ESS, and AIS scores between 2019 and 2020 within both the strong self-isolation group and no/little self-isolation group. With respect to sleep habits, earlier bedtime (*p* = 0.006) and increased sleep duration (*p* = 0.014) were found in the strong self-isolation group. The former (*p* = 0.009) was also found in the no/little self-isolation group, but we found significant differences in sleep duration between the no/little self-isolation group and the strong self-isolation group (*p* = 0.047). Therefore, self-isolation due to COVID-19 had relatively small one-year effects on depression, sleepiness, and insomnia in a clinical population.

## 1. Introduction

The severe acute respiratory syndrome coronavirus has been widely and rapidly transmitted around the world. As a result, the coronavirus disease 2019 (COVID-19) pandemic has drastically altered the life of millions of individuals worldwide. It has been reported that the COVID-19 pandemic could lead to deleterious mental health effects and other problems [1]. A systematic review of 72 reports on mental health during the COVID-19 pandemic showed that the prevalence of acute symptoms of depression and insomnia exceeded 30% [2]. Under the spread of COVID-19, uncontrolled fears associated with infection, anxiety related to job insecurity and financial concerns, reduced social interactions, and changes in sleep habits are thought to contribute to the worsening of these mental health conditions [1,3,4].

Although several studies have focused on high-risk groups of poor mental health, such as patients with mental illness, little is known about the mental health of patients with sleep disorders. For example, Zhou et al. [5] reported a 16.9% incidence of depressive symptoms and a 26.7% of insomnia symptoms among psychiatric, neurological, and sleep outpatients during the COVID-19 outbreak in China. In the context of COVID-19, patients with sleep disorders can also be considered as a risk group for poor mental health. Indeed, home confinement by COVID-19 may have effects on sleep rhythms, which play an important role in mental health conditions [1]. Furthermore, patients who already have sleep disorders are more likely to suffer from a disruption in their sleep patterns during home confinement, which may lead to poor mental health. Thus, the effects of self-isolation in this context on sleep habits and mental health status need to be investigated.

The spread of COVID-19 in Japan differs considerably from that reported by studies in other countries. A state of emergency was declared for the whole of Japan on 7 April 2020 [6]. Although people were recommended to stay at home and refrain from moving to other prefectures from 8 April to 31 May 2020 [7], home confinement for the COVID-19 was not as strictly enforced in Japan as in other countries. People could decide for themselves whether to go out (hence, it was called as “self-isolation”) or not (“lockdown”). The degree of self-restraint varied from person to person in Japan: some went out rarely, while others went to work or for shopping as usual. Similarly, some did not see anyone other than family members at all, while others went for a drink with friends. Therefore, individual differences in the degree of restrictions on going out should be considered for a better analysis of the situation in Japan. Furthermore, there has been little discussion on the relationship between changes in sleep habits and mental health conditions under the non-strict restraint of going out (i.e., self-isolation) in Japan. Although previous studies have reported that the strict home confinement (i.e., lockdown) during the pandemic affects sleep habits and mental health in infected areas, it is not clear whether the self-isolation period in Japan will adversely affect sleep habits and mental health conditions.

This study was conducted retrospectively in patients from our outpatient sleep clinic for three reasons. First, patients with prior sleep disorders might be at high risk for poor sleep habits and mental health problems in the COVID-19 context. Further, insomnia is thought to be a predictor for depression [8]. Moreover, self-reported insomnia scales have been reported to be significantly associated with depression in the Shiga prefecture, Japan, the same location as in this study [9]. Although populations with sleep problems are more likely, in principle, to have poor mental status in COVID-19 situations and should be examined, no studies have focused on patients with sleep disorders. Second, a comparison of the conditions before and after the COVID-19 epidemic was possible. We regularly recorded data on patients’ mental health and sleep habits, which allowed us to compare pre- and post-epidemic data. Sleep habits differ substantially among individuals; it is therefore crucial to identify the changes in sleep habits. Third, it was possible to determine the influence of predictors on mental health conditions during the period of self-isolation due to the retrospective nature of the study. Even if prospective studies are conducted in the future, it will be difficult to control for situational factors such as release from self-isolation and the ever-changing infection situation. Therefore, it would be useful to conduct a retrospective study to identify predictors that influence mental health conditions.

Here, we focused on the effects of sleep habits and social interactions on mental health condition during the self-isolation period due to COVID-19. Changes in sleep habits could lead to mental health problems in lockdown situations. In relation to this, Leone et al. [10] hypothesized that reduced exposure to sunlight, which maintains the biological and circadian rhythms, may disturb sleep habits and biological processes during the lockdown period. Leone et al. [10] reported that, during lockdown, delayed circadian rhythm worsened sleep quality, although sleep duration in the weekdays was prolonged, and social jetlag decreased. Reduced close social interactions and loneliness were reported to worsen depression [11,12]. We additionally considered seasonal effects as a confounding factor. Previously, we found that a preceding decrease in sunlight duration was associated with increased suicide attempts [13]. Sunlight duration changes throughout seasons, and likewise depression and suicidal rates show seasonal variations [13,14]. Hence, to minimize seasonal changes in both sleep and depression, we analyzed changes between April and July 2019 and May 2020. 

This was a retrospective study of sleep habits and mental health in patients attending the outpatient sleep clinic at Shiga University of Medical Science Hospital after self-isolation and about one year before. We conducted an exploratory study to examine the effects of changes in sleep habits and lifestyle patterns during the period of self-isolation in Japan. The present study aimed to determine the effects of the degree of self-isolation on (a) depression, sleepiness, insomnia, and sleep habits and (b) sleep habits and social interactions on depression, sleepiness, and insomnia.

## 2. Materials and Methods 

### 2.1. Participants 

**Figure 1:**
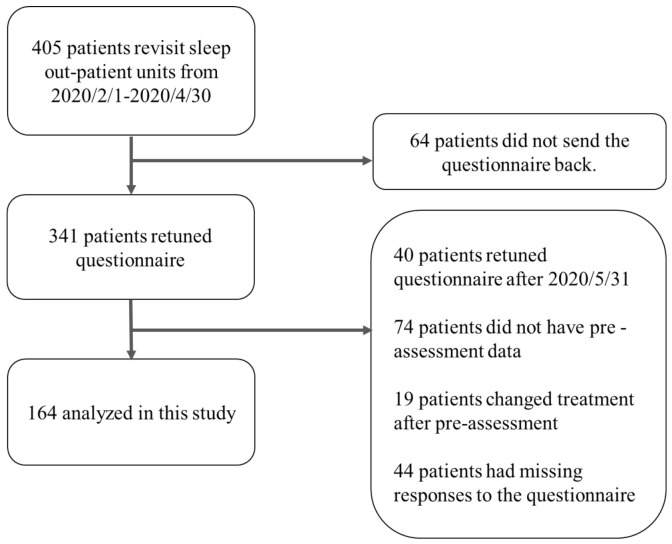
A flowchart of this study.

**Figure 2:**
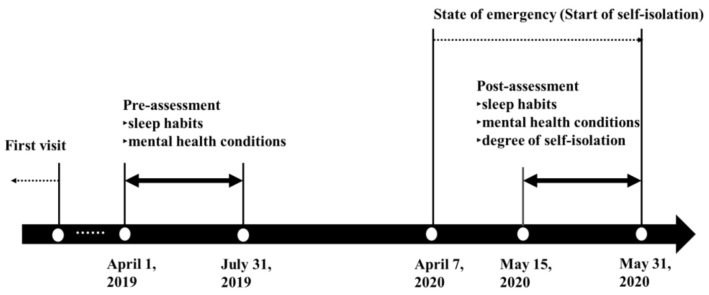
A period of self-isolation during the COVID-19 pandemic and timeline of the study.

The participants were recruited among patients who regularly visited the sleep outpatient clinic in Shiga University Medical Science Hospital. A flowchart of subject selection is shown in Figure 1, and the survey period is shown in Figure 2. We included participants who answered both pre-assessment and post-assessment questionnaires without missing responses. The pre-assessment data were acquired from medical records, which included the answers to questionnaires provided between April 2019 and July 2020. The reason why we focused on patients with pre-assessment data from April to July was to minimize the effects of seasonal fluctuation of mental health and sleep habits [13]. We excluded patients whose treatments changed between pre-assessment and post-assessment periods. The post-assessment questionnaires were mailed to the homes of 405 patients on 15 May 2020. Post-assessment data were collected from 15 May 2020 to 31 May 2020. We used data up to 31 May 2020, because we considered the impact on mental health and the loosening of self-isolation due to the end of the state of emergency. Shift workers were not included as participants in the present study.

### 2.2. Measures

#### 2.2.1. Degree of Self-Isolation 

Participants were asked to rate the change in the frequency of going out compared with the same time the previous year, on a six-point scale, with a rating of 1 being unchanged or increased; 2, slightly reduced: reduced to 80–90%; 3, fairly reduced: reduced to 60–70%; 4, reduced to about half: reduced to 40–50%; 5, considerably reduced: reduced to 20–30%; and 6, not went out: reduced to 0–10%). The participants were classified into no/little self-isolation (degrees 1, 2, 3, and 4: reduction <70%) and strong self-isolation (degrees 5 and 6: reduction ≥70%) by the frequency of going out. 

#### 2.2.2. Changes in Lifestyle

Sleep habits. The assessment of sleep habits included bedtime, wake-up time, total sleep time, and sleep onset latency. Information on these sleep habits was obtained from the patients’ self-reports.

Social interactions. The question required participants to state the degree to which they refrained from meeting with people (with the exception of family) compared with the same time the previous year on a 6-point scale, with 1 being unchanged or increased; 2, slightly reduced: reduced to 80–90%; 3, fairly reduced: reduced to 60–70%; 4, reduced to about half: reduced to 40–50%; 5, considerably reduced: reduced to 20–30%; and 6, no meeting: reduced to 0–10%. 

#### 2.2.3. Mental Health Conditions

The Patient Health Questionnaire-9 (PHQ-9). The Patient Health Questionnaire-9 (PHQ-9) is a validated 9-item self-report questionnaire that assesses the severity of depression with possible scores ranging from 0 to 27 points [15]. PHQ-9 scores of 5, 10, 15 and 20 represent thresholds demarcating the lower limits of mild, moderate, moderately severe, and severe depression, respectively. Cronbach’s α at pre-assessment and post-assessment was 0.868 and 0.872, respectively.

The Epworth Sleepiness Scale (ESS). The Epworth Sleepiness Scale (ESS) is a validated 8-item self-report questionnaire, which is used to assess the severity of insomnia with a possible score range of 0–24 points [16]. An ESS score of 11 points or higher indicates excessive daytime sleepiness. Cronbach’s α at pre-assessment and post-assessment was 0.881 and 0.824, respectively.

The Athens Insomnia Scale (AIS). The Athens Insomnia Scale (AIS) is a validated 8-item self-report questionnaire that assesses the severity of insomnia with a possible score ranging from 0 to 24 points [17,18]. An AIS score of 6 is the optimal cutoff score based on the balance between sensitivity and specificity [17]. AIS total scores in the range of 6–7 and ≥ 10 were classified as suspected and definite insomnia, respectively [9]. Cronbach’s α at pre-assessment and post-assessment was 0.843 and 0.824, respectively.

### 2.3. Statistical Analysis

SPSS software version 26.0 (IBM, Armonk, NY, USA) was employed for statistical analysis. First, we conducted a *t*-test for age and Chi-square tests for sex, diagnosis, and treatment to confirm the lack of differences between the two groups. Since not all variables had a normal distribution, nonparametric tests were employed. We conducted a Wilcoxon signed-rank test to examine changes in sleep habits and mental health conditions between 2019 and 2020 for each of the two groups. Sleep habit variables (bedtime, wake-up time, total sleep time, and sleep onset latency) were converted to numerical values and tested. The changes in AIS, ESS, PHQ-9, and sleep habits (bedtime, wake-up time, total sleep time, and sleep onset latency) were calculated as the difference between the respective post-assessment and pre-assessment scores. Finally, in order to investigate whether the interactions between changes in sleep habits and those of mental health conditions were different in the two groups, we conducted a hierarchical multiple regression analysis to examine the changes in sleep habits and social interactions on mental health in each group. We controlled the effect of age and sex. Explanatory variables were inputted by the entry input method, with age and sex in step 1, and changes in sleep habits, restraint on meeting with people, and changes in AIS, ESS, and PHQ-9 scores in step 2. 

### 2.4. Ethical Considerations

The study protocol was approved by the Ethics Committee of Shiga University of Medical Science (R2015-229). This study was conducted according to the Declaration of Helsinki (2013). Written informed consent was not obtained because of the retrospective nature of the study. We disclosed the study protocol on the website (https://rinri.shiga-med.ac.jp/rinri/publish_document.aspx?ID=273). Subjects were offered the opportunity to opt out of the study.

## 3. Results

### 3.1. Participants

The analysis included 164 patients (21 women), with a mean age of 63.60 (standard deviation [SD] = 13.54, range = 14–94) years.

A self-isolation of 0–10, 20–30, 40–50, 60–70, 80–90, and >90% was found in 17 (10.4%), 26 (15.9%), 32 (19.5%), 17 (10.4%), 44 (26.8%), and 28 (17.1%) patients, respectively (supplementary Table S1). A total of 92 and 72 subjects were allocated to the no/little self-isolation group (isolation <70%) and the strong self-isolation group (isolation ≥70%), respectively. At post-assessment, PHQ-9 ≥ 5, ESS ≥ 11, and AIS ≥ 6 were observed in 29.3, 15.9, and 28.7% of the patients, respectively. There were no significant differences in age or sex between the two groups. In both groups, nearly 95% of patients were diagnosed with obstructive sleep apnea, and nearly 93% were under continuous positive airway pressure treatment. Details on demographic data, diagnosis, and treatment of each group are shown in Supplementary Table S2. 

### 3.2. Changes in Sleep Habits, Depression, Sleepiness, and Insomnia

Table 1 shows the results of the Wilcoxon signed-rank test of sleep habits, PHQ-9, ESS, and AIS. The strong self-isolation group had a significantly earlier bedtime (Z = 11.00, *p* = 0.006) and an increase in total sleep time (Z = 37.00, *p* = 0.014) in post-assessment. Moreover, the no/little self-isolation group had a significantly earlier bedtime (Z = 20.00, *p* = 0.009) and a decrease in ESS score at post-assessment (Z = 28.00, *p* = 0.013). Comparisons between groups at post-assessment and change score are shown in Supplementary Tables S3 and S4.

### 3.3. Effects of Changes in Sleep Habits and Social Interactions on Depression, Insomnia, and Sleepiness

Table 2 represents the result of a hierarchical multiple regression analysis in the strong self-isolation group. In the strong self-isolation group, the predictive models with changes in PHQ-9, ESS, and AIS as the dependent variable were significant (PHQ-9: F (9, 62) = 7.489, *p* = 0.000; ESS: F (9, 62) = 2.268, *p* = 0.029; AIS: F (9, 62) = 10.884, *p* = 0.000). In terms of changes in PHQ-9, the significant standardized coefficients revealed changes in total sleep time (β = 0.287, *p* = 0.034) and changes in AIS (β = 0.725, *p* = 0.000). Next, with respect to ESS, significant standardized coefficients were found only for changes in AIS (β = 0.489, *p* = 0.004). Finally, for AIS, significant standardized coefficients were found for changes in total sleep time (β = -0.337, *p* = 0.005), changes in PHQ-9 (β = 0.586, *p* = 0.000), and changes in ESS (β = 0.252, *p* = 0.004).

Table 3 shows the results of a hierarchical multiple regression analysis in the no/little self-isolation group. In the no/little self-isolation group, the predictive models with changes in PHQ-9 and ESS as the dependent variables were not significant (PHQ-9: F (9, 82) = 1.156, *p* = 0.334; ESS: F (9, 82) = 0.882, *p* = 0.544). In terms of changes in AIS, conversely, the predictive model was significant (F (9, 82) = 4.263, *p* = 0.000). Significant standardized coefficients were found for sex (β = 0.213, *p* = 0.029), degree of social participation (β = 0.303, *p* = 0.003), changes in total sleep time (β = -0.417, *p* = 0.000), changes in PHQ-9 (β = 0.190, *p* = 0.048) scores, and AIS scores.

## 4. Discussion

The purpose of present study was to determine the effects of the degree of self-isolation on (a) changes in depression, sleepiness, insomnia, and sleep habits and (b) the effects of changes in sleep habits and social interactions on depression, sleepiness, and insomnia. The prevalence of PHQ-9 ≥ 5, ESS ≥ 11, and AIS ≥ 6 among our patients at post-assessment was 29.3, 15.9, and 28.7%, respectively. In a study conducted in psychiatric, neurological, and sleep outpatients [5] in China, an incidence of 16.9% was reported for patients with depressive symptoms and an incidence of 26.7% was reported for patients with insomnia symptoms. Although the prevalence of depression was slightly higher in Japan, the prevalence of insomnia was similar to results of previous studies. The prevalence of PHQ-9 ≥ 5, ESS ≥ 11, and AIS ≥ 6 in city government employees in the Shiga prefecture, Japan, in 2017 was 41.0, 25.5, and 38.2%, respectively [9]. In our clinical settings, we evaluated PHQ-9, ESS, and AIS at the first visit and every six months thereafter for early intervention. This may be why depression, sleepiness, and insomnia scores were lower in clinical settings than among city government employees. In Japan, the rate of suicide decreased in the first half (January to Jun) of 2020 but increased after July 2020 [19]. The governor recommended release from self-isolation after May 2020. A negative correlation between gross national product and suicide rates was found in Japan [20]. Therefore, it is not the self-isolation, but rather other factors, such as economic uncertainty, that may affect Japanese suicidal rates.

In this study, self-isolation did not worsen mental health conditions such as depression, insomnia, and sleepiness in either group. The PHQ-9, ESS, or AIS scores were not significantly changed in the strong self-isolation group. In the no/little self-isolation group, there was no worsening of sleep habits or mental status, and the ESS scores were rather improved (Table 3). Regardless of the intensity of self-isolation, it was surprising that there was no worsening of mental health conditions. Although this could be interpreted to mean that mental health did not markedly worsen in a country like Japan, where behavioral restraints were not very strict, we are not very optimistic about this result. Because the present study was conducted during a period of 5–8 weeks of self-isolation (self-isolation was recommended by the governor on 8 April, and our post-assessment questionnaire survey was conducted between 15 May and 31 May), we cannot analyze long-term effects of self-isolation based on the present study. Long-term self-isolation could have deleterious effects on mental health due to the disturbance of biological and circadian rhythms and changes in socio-economic conditions. It is also possible that the older age group in this study may have influenced the results. The negative psychological effects of home confinement have been shown to be greater in younger than in older populations [21]. This study also shows that poor cohabitation ratings were associated with greater negative effects, and such variables will need to be examined in the future.

In terms of sleep habits before and after self-isolation comparison, we found that patients went to bed significantly earlier in both groups. However, only the strong self-isolation group was found to have a longer sleep time during the self-isolation period compared with the previous year. Other studies have also reported increases in sleep duration during the COVID-19 lockdown [10,22,23]. We found a significant increase in total sleep time in the strong self-isolation group. This increase may be similar to that reported in other studies. However, such an increase was not found in our no/little self-isolation group. Bedtime during lockdown has been reported to be delayed [10,22,23]. We found that bedtime was moved earlier in our strong self-isolation group. We cannot clarify the reason why bedtime was shifted earlier. Sleep duration in Japan has been reported to be the shortest among OECD countries [24]. Sleep-deprived patients with a strong regime of self-isolation might use this opportunity to allow their sleep time to be better adjusted to their natural circadian preference.

Another objective of this study was to investigate the effects of self-isolation and changes in sleep habits on depression, insomnia, and sleepiness. As there was no worsening of overt mental health (Table 1), it is necessary to understand the structures that affect mental health conditions, looking ahead to a long-lasting coexistence situation with COVID-19. First, the result of a hierarchical multiple regression analysis for those under strong self-isolation indicated significant predictive models for all mental health indicators (Table 3). Particular attention should be paid to depressive symptoms. The result showed that increased sleep duration and worsening in the AIS score were associated with worsening of depression. This association was more significant in the strong self-isolation group than in the no/little self-isolation group. This relationship may be explained by the delay in circadian rhythms due to reduced daylight hours [10], where the advances in bedtime might create a gap in circadian rhythms or other such alterations. The unusual form of prolonged sleep duration may have paradoxically led to a decline in sleep quality and affected depressive symptoms. This suggests that we need to focus on sleep quality rather than sleep duration under the strong restraint of going out in order to slow down the spread of infection. In the future, it may be important to assess not only sleep duration but also objective changes in sleep quality, which can be monitored with portable devices [25]. 

Next, the result of the hierarchical multiple regression analysis in those with no/little self-isolation showed that the predictive model was significant only for changes in insomnia (Table 2). Predictive models of sleepiness and depression were not valid, and other variables were found to be involved in mental health changes in those with no/little self-isolation. This result revealed that female sex, refraining from social gatherings, and decreased sleep duration influenced the worsening of insomnia. It is noteworthy that the lifestyle parameter was linked to the worsening insomnia even among those with no/little self-isolation. This result may be explained by the effect of loneliness on insomnia. For example, Voitsidis et al. [26] found that loneliness was a risk factor for insomnia symptoms under the COVID-19 epidemic situation. A recent meta-analysis has shown that the association between loneliness and insomnia symptoms tends to be stronger in the elderly and in men [12]. Although it remains to be examined whether the findings of the current sample in older subjects, including the male predominance, are replicated in other populations, attention should be focused on whether the restrictions on social gatherings increase loneliness and negatively influence insomnia, even without strong social distancing restrictions. 

Finally, this study had some limitations. First, the number of cases and deaths due to COVID-19 has been lower in Japan than in other countries, and thus, this result may not be applicable to countries with a high spreading rate. In Japan, it may have been possible to detect the impact of lifestyle on mental health in the COVID-19 situation, because the impact on social conditions was smaller than it might be in other countries. However, the present study may have implications for future interventions in a society where the impact of social changes associated with infection has subsided and the impact of individual habits on mental health has increased. Second, to our knowledge, this is the first report on COVID-19-related mental health issues in Japan; hence, our results cannot be compared with those of other studies in Japan. Third, we included patients with sleep disorders in this study to detect changes by comparing the data at two time points; as a result, participants were mainly consisting of elderly subjects. There was a gender disparity in present study (Table S2). The majority of outpatients who regularly visit our outpatient clinic had obstructive sleep apnea, which is a male predominant disorder (Table S2). Therefore, future work should examine whether our results are applicable to healthy young people and other populations. Fourth, the post-assessment time was about two months after the state of emergency, and the self-isolation period might have been too short. Although the results of this study cannot necessarily explain the long-term effects of self-isolation, it is important to show that changes in sleep habits even during a short period of self-isolation adversely affect mental health. Further studies are needed in order to investigate the long-term effects of self-isolation.

Despite these limitations, the present study provides significant insights in two respects. First, it is the first to investigate changes in sleep habits and mental health under self-isolation. Second, the risk factors for the worsening of mental health are clarified in relation to the degree of self-isolation. In particular, we found a high risk for poor mental health not only among those with strong self-isolation, which has been the focus of previous studies, but also among those with a lower degree of self-isolation. As COVID-19 progresses toward convergence, it is expected that other countries will transition to a regime of self-isolation similar to the one followed in Japan. Therefore, further research on the degree of self-isolation will contribute to supporting mental health in societies living with infections.

## 5. Conclusions

In summary, we found that self-isolation due to COVID-19 did not worsen mental health conditions such as depression, sleepiness, and insomnia. A change in sleep habits was observed only in the strong self-isolation group. This study was conducted only two months after the beginning of the self-isolation period; nevertheless, the different relationships between sleep habits and mental health conditions in each group were still observable. Since the current situation in Japan could be a reference model for society after overcoming the acute phases of the COVID-19 pandemic, looking ahead to a long-lasting period of coexistence with COVID-19, our results have clinical implications that may remain even after the pandemic subsides.

## Figures and Tables

**Table 1:** Result of Wilcoxon signed-rank test on sleep habits and Patient Health Questionnaire-9 (PHQ-9), Athens Insomnia Scale (AIS), and Epworth Sleepiness Scale (ESS).

Variables	Pre		Post		Z	*p*-Value
	Median		Median			
No/little self-isolation (*N* = 92)						
Wake up time (hh:mm)	6:00	(5:00–6:30)	6:00	(5:05–7:00)	29.00	0.938
Bedtime (hh:mm)	23:00	(22:00–24:00)	23:00	(22:00–24:00)	20.00	0.009
Total sleep time (hh:mm)	6:00	(5:07–7:30)	6:30	(5:37–7:30)	37.00	0.749
Sleep onset latency (min)	10	(5–20)	10	(5–20)	33.00	0.460
PHQ–9	2.00	(1.00–5.00)	3.00	(0.25–6.00)	36.00	0.540
ESS	6.00	(4.00–8.75)	6.00	(3.00–8.00)	28.00	0.013
AIS	3.00	(1.00–6.00)	3.00	(2.00–6.00)	42.00	0.249
Strong self-isolation (*N* = 72)						
Wake up time (hh:mm)	6:10	(5:22–7:15)	6:20	(5:30–7:15)	25.00	0.713
Bedtime (hh:mm)	23:30	(22:00–24:30)	23:00	(22:00–24:00)	11.00	0.006
Total sleep time (hh:mm)	6:00	(5:30–7:00)	6:30	(5:30–7:30)	37.00	0.014
Sleep onset latency (min)	10	(5.5–7.5)	10	(8.2–30)	26.00	0.306
PHQ-9	2.00	(1.00–6.75)	2.00	(0.25–5.00)	21.00	0.057
ESS	6.00	(4.00–11.00)	6.00	(4.00–8.75)	26.00	0.202
AIS	4.50	(2.00–6.00)	4.00	(2.00–6.00)	29.00	0.441

AIS = Athens Insomnia Scale, ESS = Epworth Sleepiness Scale, PHQ-9 = Patient Health Questionnaire. Numbers in parentheses represent the first to third quartiles.

**Table 2:** Results of multiple regression analysis in the strong self-isolation group.

Variables	Changes in PHQ-9				Changes in ESS				Changes in AIS			
	Step 1	*p*-Value	Step 2	*p*-Value	Step 1	*p*-Value	Step 2	*p*-Value	Step 1	*p*-Value	Step 2	*p*-Value
Age	0.262	0.025	0.171	0.068	0.009	0.938	0.023	0.846	0.171	0.144	−0.025	0.770
Sex	−0.197	0.091	−0.002	0.987	−0.169	0.168	−0.077	0.526	−0.241	0.041	−0.125	0.151
Degree of social participation			−0.100	0.287			−0.145	0.216			0.079	0.352
Changes in wake-up time			−0.037	0.736			0.045	0.740			0.092	0.347
Changes in bedtime			0.145	0.171			0.059	0.660			−0.149	0.119
Changes in total sleep time			0.287	0.034			0.280	0.101			−0.337	0.005
Changes in sleep onset latency			0.069	0.588			−0.200	0.210			0.106	0.356
Changes in PHQ-9			-	-			−0.224	0.158			0.586	0.000
Changes in ESS			−0.143	0.158			-	-			0.252	0.004
Changes in AIS			0.725	0.000			0.489	0.004			-	-
R^2^	0.127	0.009	0.521	0.000	0.029	0.361	0.248	0.029	0.103	0.023	0.612	0.000
ΔR^2^			0.394	0.000			0.219	0.021			0.509	0.000

AIS = Athens Insomnia Scale, ESS = Epworth Sleepiness Scale, PHQ-9 = Patient Health Questionnaire. Step 1: sex (0 = men, 1 = women), age; step 2: degree of social participation (0 = unchanged or increased to 6 = never met people), changes in wake-up time, bedtime, total sleep time, sleep onset latency, PHQ-9, ESS, and AIS.

**Table 3:** Results of multiple regression analysis in the no/little self-isolation group.

Variables	Changes in PHQ-9				Changes in ESS				Changes in AIS			
	Step 1	*p*-Value	Step 2	*p*-Value	Step 1	*p*-Value	Step 2	*p*-Value	Step 1	*p*-Value	Step 2	*p*-Value
Age	0.060	0.571	0.002	0.989	0.139	0.192	0.131	0.242	0.217	0.038	0.093	0.334
Sex	0.018	0.866	−0.059	0.599	0.047	0.657	0.055	0.631	0.163	0.116	0.213	0.029
Degree of social participation			−0.058	0.626			0.095	0.426			0.303	0.003
Changes in wake-up time			0.097	0.377			−0.054	0.630			−0.151	0.114
Changes in bedtime			−0.096	0.386			0.060	0.593			−0.025	0.798
Changes in total sleep time			0.131	0.291			0.093	0.459			−0.417	0.000
Changes in sleep onset latency			0.122	0.270			−0.155	0.166			0.024	0.803
Changes in PHQ-9			-	-			0.173	0.121			0.190	0.048
Changes in ESS			0.168	0.121			-	-			0.008	0.937
Changes in AIS			0.247	0.048			0.010	0.937			-	-
R^2^	0.004	0.846	0.113	0.334	0.020	0.405	0.088	0.544	0.067	0.047	0.319	0.000
ΔR^2^			0.109	0.202			0.068	0.529			0.252	0.000

AIS = Athens Insomnia Scale, ESS = Epworth Sleepiness Scale, PHQ-9 = Patient Health Questionnaire. Step 1: sex (0 = men, 1 = women), age; step 2: degree of social participation (0 = unchanged or increased to 6 = never met people), changes in wake-up time, bedtime, total sleep time, sleep onset latency, PHQ-9, ESS, and AIS.

## Supplementary Materials

The following items are available online at https://www.mdpi.com/1660-4601/17/23/8971/s1, Table S1: Frequency distribution of the restrictions on outing and meeting people, Table S2: Demographic data, diagnosis, and treatment in the no/little self-isolation group and the strong self-isolation group. Table S3: Result of Mann–Whitney U test of sleep habits, PHQ-9, ESS, and AIS during COVID-19. Table S4: Result of Mann–Whitney U test of changes in sleep habits, PHQ-9, ESS, and AIS.
