# Supplementary material for: Self-Isolation Due to COVID-19 Is Linked to Small One-Year Changes in Depression, Sleepiness, and Insomnia: Results from a Clinic for Sleep Disorders in Shiga Prefecture, Japan
Source: Int J Environ Res Public Health. 2020 Dec 2;17(23):8971. doi: 10.3390/ijerph17238971 (PMC7730558; doi:10.3390/ijerph17238971)
Supplement: Supplementary file 1 [file ijerph-17-08971-s001.pdf]

Supplementary Material

Table S1. Frequency distribution of the restrictions on outing and meeting people.

		Restrictions on Outing						
		**1**	**2**	**3**	**4**	**5**	**6**	**Sum**
Restrictions on meeting people	1	11	5	3	3	3	0	25
	2	3	12	5	1	2	0	23
	3	1	7	8	1	2	1	20
	4	0	2	6	4	3	2	17
	5	2	0	7	5	22	4	40
	6	0	0	3	3	12	21	39
	Sum	17	26	32	17	44	28	164

Answers: 1 = unchanged or increased (no reduction); 2 = slightly reduced (reduced to 80–90%); 3 = fairly reduced (reduced to 60–70%); 4 = reduced to about half (reduced to 40–50%); 5 = considerably reduced (reduced to 20–30%); 6 = not went out or no meeting with people (reduced to 0–10%).

Table S2. Demographic data, diagnosis, and treatment in the no/little self-isolation group and the strong self-isolation group.

	No/Little Self-Isolation (*N* = 92)		Strong Self-Isolation (*N* = 72)		*p*
Demographic data					
age at post-assessment	63.47	± 13.09	63.78	± 14.19	.661
sex (female)	81	(11)	62	(10)	.445
Diagnosis					.622
Sleep related breathing disorders	88	(95.7%)	68	(94.4%)	
Insomnia	1	(1.1%)	0	(0%)	
Central disorders hypersomnolence	1	(1.1%)	2	(2.8%)	
Circadian rhythm sleep-wake disorders	1	(1.1%)	0	(0%)	
Sleep related movement disorders	1	(1.1%)	1	(1.4%)	
Others	0	(0%)	1	(1.4%)	
Treatment					.608
Continuous Positive Airway Pressure	86	(93.5%)	67	(93.1%)	
Adaptive servo ventilation	1	(1.1%)	0	(0%)	
Oral appliance	1	(1.1%)	1	(1.4%)	
Anti-insomnia drug	2	(2.2%)	1	(1.4%)	
Modafinil	1	(1.1%)	2	(2.8%)	
Dopamine agonist	1	(1.1%)	0	(0%)	
Follow-up	0	(0%)	1	(1.4%)	

99.4% of patients with sleep-related breathing disorders were obstructive sleep apnea.

Table S3. Result of Mann-Whitney U test of sleep habits, PHQ-9, ESS and AIS during COVID-19.

	**Total**		**No/Little**		**Strong**		***U***	***p*-value**
N	164		92		72			
Wake -up time (hh:mm)	6:00	(5:30to7:00)	6:00	(5:05to7:00)	6:20	(5:30to7:15)	3648.00	.264
Bedtime (hh:mm)	23:00	(22:00to24:00)	23:00	(22:00to24:00)	23:00	(22:00to24:00)	3481.50	.572
Total sleep time (hh:mm)	6:30	(5:30to7:30)	6:30	(5:37to7:30)	6:30	(5:30to7:30)	3373.50	.837
Sleep onset latency (min)	10	(5.6to20)	10	(5to20)	10	(8.2to30)	3520.50	.482
PHQ-9	3.00	(0.25to5.00)	3.00	(0.25to6.00)	2.00	(0.25to5.00)	3117.00	.513
ESS	6.00	(3.00to8.00)	6.00	(3.00to8.00)	6.00	(4.00to8.75)	3646.00	.266
AIS	4.00	(2.00to6.00)	3.00	(2.00to6.00)	4.00	(2.00to6.00)	3646.00	.265

AIS = Athens Insomnia Scale, ESS = Epworth Sleepiness Scale, PHQ-9 = Patient Health Questionnaire. No/Little self-isolation: outings ≥30% compared with one years before, Strong self-isolation: outings <30% compared with one years before. Numbers in parentheses represent the first to third quartiles.

Table S4. Result of Mann-Whitney U test of changes in sleep habits, PHQ-9, ESS and AIS.

	**Total**		**No/Little**		**Strong**		***U***	***p*-value**
N	164		92		72			
Changes in Wake -up time (hh:mm)	0:00	(-0:30to0:30)	0:00	(-0:30to0:17)	0:00	(-0:26to0:30)	0.22	.825
Changes in Bedtime (hh:mm)	0:00	(-0:30to0:00)	0:00	(-0:30to0:00)	-0:10	(-0:45to0:00)	-1.11	.267
Changes in Total sleep time (hh:mm)	0:00	(-0:30to0:30)	0:00	(-0:30to0:30)	0:15	(-0:25to0:57)	1.83	.068
Changes in Sleep onset latency (min)	0	(-2to5)	0	(-2to5)	0	(0to5)	0.41	.685
Changes in PHQ-9	0.00	(-2.00to1.00)	0.00	(-1.00to1.75)	0.00	(-2.00to1.00)	-1.86	.062
Changes in ESS	0.00	(-2.00to1.00)	-1.00	(-2.00to1.00)	0.00	(-2.75to2.00)	0.50	.614
Changes in AIS	0.00	(-1.00to1.00)	0.00	(-1.00to2.00)	0.00	(-2.00to1.00)	-1.35	.178

AIS = Athens Insomnia Scale, ESS = Epworth Sleepiness Scale, PHQ-9 = Patient Health Questionnaire. No/Little self-isolation: outings ≥30% compared with one years before, Strong self-isolation: outings <30% compared with one years before. Numbers in parentheses represent the first to third quartiles.
